# Understanding the role of lady health workers in improving access to eye health services in rural Pakistan – findings from a qualitative study

**DOI:** 10.1186/s13690-021-00541-3

**Published:** 2021-02-17

**Authors:** Stevens Bechange, Elena Schmidt, Anna Ruddock, Itfaq Khaliq Khan, Munazza Gillani, Anne Roca, Imran Nazir, Robina Iqbal, Sandeep Buttan, Muhammed Bilal, Leena Ahmed, Emma Jolley

**Affiliations:** 1 Sightsavers Pakistan Country Office, Plot 3-A, Street 7, Sector G-10/2, Islamabad, Pakistan; 2 Sightsavers - United Kingdom, Haywards Heath, UK; 3 Sightsavers – India, New Delhi, India

**Keywords:** Access to eye health services, Community health workers, Patient compliance with referrals, Qualitative methods, Pakistan

## Abstract

**Background:**

In 1994, the Lady Health Workers (LHWs) Programme was established in Pakistan to increase access to essential primary care services and support health systems at the household and community levels. In Khyber Pakhtunkhwa (KPK) province in northern Pakistan, eye care is among the many unmet needs that LHWs were trained to address, including screening and referral of people with eye conditions to health facilities. However, despite an increase in referrals by LHWs, compliance with referrals in KPK has been very low. We explored the role of LHWs in patient referral and the barriers to patient compliance with referrals.

**Methods:**

Qualitative methodology was adopted. Between April and June 2019, we conducted eight focus group discussions and nine in-depth interviews with 73 participants including patients, LHWs and their supervisors, district managers and other stakeholders. Data were analysed thematically using NVivo software version 12.

**Results:**

LHWs have a broad understanding of basic health care and are responsible for a wide range of activities at the community level. LHWs felt that the training in primary eye care had equipped them with the skills to identify and refer eye patients. However, they reported that access to care was hampered when referred patients reached hospitals, where disorganised services and poor quality of care discouraged uptake of referrals. LHWs felt that this had a negative impact on their credibility and on the trust and respect they received from the community, which, coupled with low eye health awareness, influenced patients’ decisions about whether to comply with a referral. There was a lack of trust in the health care services provided by public sector hospitals. Poverty, deep-rooted gender inequities and transportation were the other reported main drivers of non-adherence to referrals.

**Conclusions:**

Results from this study have shown that the training of LHWs in eye care was well received. However, training alone is not enough and does not result in improved access for patients to specialist services if other parts of the health system are not strengthened. Pathways for referrals should be agreed and explicitly communicated to both the health care providers and the patients.

## Background

In many low- and middle-income countries (LMICs), including Pakistan, access to healthcare services is often limited, particularly in remote rural locations and for disadvantaged population groups, such as older people, women, people from poor households and those with disabilities [1–3]. In 1994, as part of a national strategy to increase access to essential primary care services, the Government of Pakistan launched the Lady Health Workers (LHWs) Programme, which aimed to strengthen health systems at the household and community levels and to connect local communities with hospital-based services [4–6]. Since then, the programme has been rolled out across the country with more than 110,000 LHWs recruited, trained and deployed to carry out monthly household visits to advise on health promotion activities, screen, provide basic treatments, refer and encourage the uptake of referrals. One of the key strengths of the programme is that LHWs are salaried and fully recognised as part of the health care workforce. They are also recruited from the local communities and thus can deliver services in a culturally appropriate manner [6–10].

Lady health workers serve the whole community, but they play a particularly important role in maternal and child health in rural and urban slum communities by coordinating efforts with traditional birth attendants and midwives, and by ensuring that all mothers and children receive adequate care [11, 12]. LHWs conduct home visits to monitor vital signs (body temperature, blood pressure, pulse and breathing rate) and provide health education, health promotion and referrals to other health services. Support for immunisation, nutrition, family planning and polio eradication has also been a major focus of their work in recent years.

Each LHW is assigned to one or two villages (usually where they live) with a population of approximately 1500 people. The LHW’s office is her house. A lady health supervisor (LHS) oversees the work of 20–25 LHWs on a routine basis, with her office typically located within a Basic Health Unit (BHU) or a Rural Health Centre (RHC), which are the first-level healthcare facilities in Pakistan. A District Programme Implementation Unit (DPIU) coordinator oversees the work of all LHSs and LHWs in a district [6, 13].

The national blindness and visual impairment survey conducted in Pakistan in 2004 estimated the all age prevalence of blindness at 0.9% [14]. The major causes of avoidable blindness were cataracts (51.5%), corneal opacity (11.8%) and refractive error/aphakia (8.6%). Other causes of visual impairment also included glaucoma, retinitis pigmentosa, optic atrophy and senile changes [14–16].

Many international non-governmental organisations (iNGOs) are working in partnership with the Government of Pakistan and other national stakeholders to improve eye care services across the country. In 2016, Sightsavers, in collaboration with the Fred Hollows Foundation (FHF) and Pakistan Institute of Community Ophthalmology (PICO), started the project “New Vision for Eye Health in Khyber Pakhtunkhwa (KPK) Province of Pakistan”. The four-year project has been implemented in four districts (Swat, Swabi, Mansehra and Haripur) in KPK with the aim to strengthen eye health services and improve the demand for and supply of eye care.

A recent Rapid Assessment of Avoidable Blindness (RAAB) conducted in Swabi and Mansehra districts estimated the prevalence of blindness among people aged 50+ years at 4.5 and 1.8%, respectively [17]. In Swabi, women carried a disproportionately high burden of eye diseases with the prevalence of blindness among women at 6.5% compared to 2.0% among men. Cataract and refractive error were the most common causes of blindness and visual impairment in both districts. Cataract surgical coverage (at visual acuity, VA < 3/60) in Swabi was 75% among women compared to 94% among men. The difference was particularly striking in older groups aged 70+ years. The proportion of poor post-surgical visual outcomes in both districts was high (26.6%), well above the World Health Organization (WHO) target of 5% [17].

To address the problem of unmet eye health needs in the focus districts, the ‘New Vision for Eye Health in Khyber Pakhtunkhwa (KPK) Province of Pakistan’ project, set out to train LHWs to screen and refer people with eye conditions to nearby eye health facilities. However, despite an increase in referrals by LHWs, the uptake of referrals was low, estimated at around 15–20% from routine programme data. As part of the project, this study set out to explore (1) the role and experiences of LHWs in eye health screening and referrals, and (2) the factors determining patient eye health seeking behaviour and uptake of referrals. This study intends to inform the Ministry of Health on the organisation and delivery of eye care services, and assist NGOs on how best to support the training of community health workers in eye care in comparable settings.

## Methods

### Study design

This qualitative study using focus group discussions (FGDs) and in-depth interviews (IDIs) was part of a project aimed at strengthening eye health services and improving the demand and supply of eye care. The project set out to train community health workers, develop referral pathways and strengthen partnerships for service delivery. Briefly, the training was delivered in each district in two stages. The first stage focused on the skills of 124 lady health worker supervisors, as master trainers in eye care programming. This training of trainers’ workshop was delivered over three days by a community ophthalmologist contracted by the project, with the support of officials from the relevant government departments in each district. At the second stage, one-day training sessions comprising 20–25 LHWs were delivered by the trained LHSs to a total of 3009 LHWs across the four project focus districts.

### Study setting and participants

FGDs were conducted with patients and LHWs from Swabi and Mansehra districts. IDIs were conducted with patients, lady health supervisors (LHSs) and government officials from the LHW provincial programme and the social welfare departments of the two districts. IDIs were used to explore themes generated from the FGDs in greater depth. Participants were interviewed about the organisation and delivery of eye care services; the role and experiences of LHWs in eye health screening and referrals; and the factors determining patient eye health seeking behaviour and the uptake of referral.

A diverse group of participants that included patients, LHWs and government officials was purposively selected to capture a range of opinions and experiences. Patients were selected to ensure diversity in terms of gender, age, geography, and history of referrals. A total of 75 participants were approached and 73 (52 women and 21 men) agreed to participate in eight FGDs and nine IDIs. On one occasion, a household member (daughter) was present during the in-depth interview. Besides a research team observer who sat in to manage the audio recording equipment and examine issues of group dynamics, no other non-participants were present during the FGDs. Two female participants declined participation in the FGDs, as they needed permission from their husbands, which they could not obtain ahead of the group discussion.

### Data collection and analysis

Face-to-face in-depth interviews and FGDs took place between April and June 2019. All interviews and group discussions were conducted by two M.Sc-level qualitative researchers (IN, male and RI, female), who were independent of the project, speaking Urdu or Pashto. Interviews took place at the district hospitals, patients’ homes or government offices and, on average, each lasted one hour. Prior to each interview or group discussion, the researchers introduced themselves and explained the study. Participants had an opportunity to ask questions and all provided consent. The two data collectors met regularly during the fieldwork to discuss emerging themes and receive feedback from the research supervisors.

A topic guide was developed based on the literature to address a broad set of themes: (1) LHWs’ training; (2) their role in the delivery of eye care; (3) LHWs’ capacity and motivation; and (4) community opportunities and challenges in accessing eye care services. English and Urdu or Pashto versions of the interview guides were reviewed after pre-testing to ensure questions were accurately translated, understandable, and followed a logical flow. The core questions were the same for all participants, while specific probes were added to explore the themes emerging from the interviews in depth. Data was collected until the point of saturation, that is until no new information or themes could be identified from the additional interviews.

IDIs and FGDs were audio-recorded, transcribed verbatim and translated into English for data analysis. Two bilingual members of the study team (IKK, LA) independently reviewed the transcripts against the original audio-recordings to ensure the quality of transcribing and translation. The transcripts were analysed thematically using both deductive and inductive approaches to explore themes and data patterns [18]. Two researchers (RI, IN) coded the first set of transcripts independently, while a senior researcher (SB) undertook coding of a subset of transcripts and met with the two main coders to discuss emerging themes and discrepancies. Similarities and differences in the coding were discussed, and the coding framework was refined, agreed and applied to the remaining transcripts. The codebook had two top-level codes: training and integration of LHWs in the eye health workforce, and patient health-seeking behaviour and barriers to referral uptake. Organizing the analyses around these top-level codes allowed the data to be blended easily with the research questions and other existing empirical work. Further sub-coding of categories within each top-level code came next. The sub-coding step assigned several new codes that (i) described the nature of the training and the health system context in which LHWs were being deployed, and (ii) captured a range of factors and challenges affecting the pathways through which patients access specialist eye care services. All coding and data analysis were conducted using NVIVO software version 12 (QSR International). The transcripts were not returned to the participants for comments. However, data analysis began during data collection through regular team meetings and reflection. Ongoing discussions between the investigators and the programme staff helped to ensure that the themes and subthemes developed accurately represented what participants conveyed during the in-depth interviews and FGDs.

### Ethical considerations

The study was reviewed and approved by the Institutional Review Board of the Pakistan Institute of Community Ophthalmology (PICO) (protocol #073/2019). All participants provided written informed consent. Participants were served with refreshments and paid an equivalent of US $3.00 to compensate for their time and transport. All identifiable information was excluded from the transcripts and data confidentiality was protected throughout the study.

## Results

Participant characteristics are described in Table 1. Among 73 participants who agreed to take part in the study, 52 were women and 21 were men. Thirty-two participants were LHWs; 35 were patients and six were government officials or healthcare managers. Among 35 patients interviewed, 17 were women and 18 were men.

**Table 1:** Participant characteristics (*n* = 73)

Characteristic	LHWs (***n*** = 32)		Officials/managers (***n*** = 6)	
Age in years, mean (range)	41	31-51	45	34-56
**Sex, n (%)**				
Female	32	100	3	50
Male	0	0	3	50
**Marital status, n (%)**				
Married	21	66	4	66
Not married	11	34	2	34
**Education level, n (%)**				
Below grade 10	29	91	0	0
Grade 10 and above	3	9	6	100
**Main source of income, n (%)**				
Salary	20	62	5	83
Remittances (husband, brother)	12	38	1	17
Other	0	0	0	0
**Time spent as LHW, n (%)**				
Less than 10 years	2	6	Not applicable	
10–20 years	21	66	Not applicable	
Above 20 years	9	28	Not applicable	
**Years in role, mean (range)**	11	4–24	18	12–30

The findings that emerged from the interviews are organised in two overarching themes: (i) integration of LHWs in the eye health workforce, and (ii) patient health-seeking behaviour and barriers to referral uptake. The first theme describes the nature of the training and the health system context in which LHWs are being deployed. The second theme captures a range of factors affecting the pathways through which patients access specialist eye care services.

### Integration of LHWs in the eye health workforce

#### Content and scope of training

Lady health workers and their supervisors described the training they had received as impactful and transformational for their practice. Prior to the training, LHWs could only listen to the complaints of people with eye issues; they were unable to take any action. The training had improved their knowledge and skills and they could now identify eye conditions and either manage the minor ones at the community level or refer patients to the hospital. As one LHW explained:

*“…[they] explained [during the training] different parts of the eye, symptoms of different diseases … and what can be done before the patient is referred to the doctor. It was also explained how to … diagnose an issue, how to clean... [the eye] and how to bandage it, if required. The use of Polyfax [eye ointment] was also explained.” [Lady Health Worker 08, Swabi]*

Another LHW said:

*“I learnt about … the eyelid, when children rub their eyes, eye lids get damaged, since they are very sensitive. We also learnt different methods for its management.” [Lady Health Worker 22, Mansehra]*

LHWs also said that they had been introduced to a general signs and symptoms’ checklist for the detection of cataracts and taught that cases should be treated immediately. They further explained that they often observed patients delaying seeking treatment for cataracts, but the training had equipped them with the skills to talk to the patients and encourage them to seek treatment as a matter of urgency.

A number of LHWs, however, said that during the training, they had limited opportunities to share their experiences, clarify project expectations or learn from each other. Some LHWs also felt that their supervisors trained as master trainers lacked direct experience of eye care programming and could not adapt the content of the training to the LHWs’ working environment in the community. In the words of one LHW:

*“Madam there is a difference of learning from a trained professional doctor and a LHS. An eye doctor has more experience and can guide us better. We would want an eye specialist to educate us … a LHS tells us the management of the problem whereas a doctor would tell us the treatment … LHS tells what to suggest, doctor will tell what to do. We believe a doctor can guide us better step by step. … The message does not remain the same when it rotates among different people. A direct source and an indirect source [of information] are two different things.” [Lady Health Worker 04, Mansehra]*

Two, male senior managers, on the other hand, felt that LHSs were best placed to train LHWs. They saw LHWs as people with limited education, which in their opinion, made it difficult for LHWs to follow or benefit from a training by medical doctors. They argued that although doctors were best placed to explain the nature of different eye diseases and treatments, they were not good trainers for people with minimal education (eight to ten years of schooling in the case of LHWs).

A key issue highlighted by all the LHWs interviewed was refresher courses. LHWs recommended that the refresher training for eye care should be carried out on a regular basis in the same way as the refresher training for other conditions managed by LHWs:

*“We get refresher trainings for other disease programmes … [every] 6 months to 2 years. Knowledge is updated, motivation is enhanced. [We] are learning new things and get involved in the field level activities. It is a good way of reminding [us of] our duties.” [Lady Health Worker 11, Swabi]*

LHWs recommended that the refresher courses should cover the topic of compliance with referrals because at present LHWs were trained to refer patients but not to ensure referral uptake. They also wanted more information about the adverse consequences of poor eye health or untreated eye conditions. They explained that the training covered well various eye diseases and how to prevent them but paid little attention to the impact of non-compliance with treatment.

It was further explained that the standard refresher courses were supported by the national LHWs programme and carried out by district health offices whenever there was a new campaign related to the areas covered by LHWs. These refresher trainings were paid for by individual disease control programmes as there was no central pot of funds to support the training. Although the primary eye care module was included in the LHW curriculum, the new knowledge would be difficult to maintain without the fully funded refresher courses.

### Availability of equipment and supplies

Following the training, all LHWs were given a PEC kit, which included a PEC manual, an E card to test visual acuity, a measuring tape, a flashlight or a torch with batteries, bandages, eye ointment and referral slips. There were two types of vision testing charts available to the LHWs, a small chart to carry with them in the community and a large chart to use in their office/house. The small (3-m) chart was provided by the project; the large (6-m) chart was provided in some districts by the national programme for the prevention of blindness.

Most LHWs found the PEC manual practical and useful for the assessment of eye health issues at the community level and specifically, identification of people with uncorrected refractive errors (UREs):

*“When we have this book [primary eye care manual] with us, then we compare different conditions with those mentioned in the book and can guide people properly... If there is something that we can deal with, then we take care of it. Otherwise we refer them for a proper treatment.” [Lady Health Worker 04, Mansehra]*

Several LHWs, however, said that they had not used their PEC kit for at least 2 years; and some kept their kits unopened as they had never used them. There was no explanation during the interviews with the LHWs why this was the case, but the district managers interviewed attributed it to the lack of clarity about the LHWs’ role and what was expected from them, which led to the reluctance of some LHWs to carry out eye care services.

The LHWs who had used their kits complained about the shortage of referral slips, flashlights/torches, batteries, eye medicines and pads. In some kits received during the training, the flashlight was missing or damaged. The consumables were difficult to replace, as the project had provided them to the LHWs only once at the beginning of the project and in limited quantity. LHWs suggested that the procurement of eye care consumables should be integrated within the LHW programme procurement systems to ensure an uninterrupted supply.

### Application of new knowledge and skills in practice

While most LHWs described the new knowledge and skills they had acquired during the training, only a few referred to the application of these in practice. Although many LHWs were generally happy about the new opportunities to provide eye related services, most of them paid more attention to other duties and health issues, i.e., maternal and child health or polio eradication campaigns because these were the areas prioritised by the government.

For those who shared their experiences, there were some apparent tensions between the desire of LHWs to identify and treat minor eye issues and the need for referrals to the upper-level facilities. Some LHWs for example, wanted to be supplied with basic consumables and drugs to manage minor eye cases in the community. Others, however, referred all patients irrespective of how minor their condition was:

*“Previously we were not sending this high number of patients to the hospital. After the training, we refer most … people to the hospital, even patients with minor problems are being referred.” [Lady Health Worker 05, Mansehra]*

At the community level, the majority of community members praised LHWs for their work in maternal and child health and polio eradication. Their work in eye health, however, raised doubts because eye health was thought to be complex and some people were hesitant as to whether LHWs were competent enough to correctly identify, manage or refer patients with eye diseases.

### Referral process

After the training, all LHWs were provided with referral slips, which they were explicitly instructed to issue to all individuals with vision or eye health problems. The referrals could be made to public or private not-for-profit facilities at the primary and secondary levels. Each referral slip contained a serial number and space to enter the date of the referral, name of the patient, sex, address and contact number of the patient, the type of eye problem identified, name of the referring LHW and the referral facility. There were three copies of each referral slip, one for the patient, one for the health facility and one for the LHW. Each LHW maintained the record of the patients referred.

The key concern expressed by the LHWs during the interviews was that their referral slip was not recognised or accepted by many facilities they referred patients to. Patients who had been referred and had visited a hospital explained how the hospital staff simply discarded their referral slip. LHWs felt that some hospital staff had no respect for LHWs and the patients they referred. They also felt that this type of behaviour damaged their reputation and the trust community members had in their work. Many found it demotivating. This is how one LHW explained her frustration:

*“[…] they would put our referral … into the dust bin... Patients do not feel good when our referrals …are put into the dust bin before their eyes...” [Lady Health Worker 20, Swabi]*

Similar views were shared by a lady health supervisor:

*“They do not give any importance to the referral slip; many times, our workers accompany their patients, but they [the hospital] say that we do not need this piece of paper.” [Lady Health Supervisor]*

LHWs felt that to change the situation, the project had to talk to the referral hospitals as well as to a partner not-for-profit eye hospital, whose staff would sign the referral slips on behalf of the LHWs to make them more legitimate.

About half of the LHWs further reported that they had believed that the patients referred by them would get some privileges at the hospital, for example that they would be prioritised in the hospital queue. They felt that as LHWs were highly respected in their communities, their referral slip would carry a special value (or “power”) at the secondary-level facility. Patients referred by the LHWs also had these impressions. However, when they visited a hospital, they did not receive any special privileges. They had to wait in a queue in the same way as any other patient. For some this was disappointing and discouraging. Some patients, notably women who had waited an entire day in the queue, shared their frustrations with other community members and discouraged them from taking up LHWs’ referrals. It was unclear from the interviews whether the expectations of special treatment by hospitals were due to past experience with other disease programmes, the role the LHWs played in the communities or because their eye care work was supported by an international development project. However, given the existing health system and social hierarchies, it seems unlikely that LHWs would expect preferential treatment for their referrals solely based on their status in the community.

### Remuneration and working conditions

Managers at the district level described the role of LHWs as a ‘bridge’ linking patients with health care facilities. They praised LHWs for their commitment and hard work, where they had to move from house to house daily:

*“LHWs are such an asset that we can utilise them anywhere. They help us in campaigns, different problems of the eye, polio, in everything.” [Manager at the district, Mansehra]*

However, study participants also recognised that the LHWs’ capacities were stretched; they were often overburdened and overworked. Most LHWs felt that any health problem in the community was ‘*dumped*’ on them and the same fixed number of LHWs was expected to do them all with no additional compensation or operational support.

*“[…] what is Rs 18,000 [US $112] per month and we remain in the field till late; and when we return home, it takes us many more hours to prepare the report and then people would come to our homes for check-ups, examinations. So, we have long hours of working and look at what we get.” [Lady Health Worker 30, Swabi]*

Some LHWs argued that the eye care programme increased their paperwork and required additional visits to the villages to remind people about hospital referrals. Some said that the monitoring visits organised specifically by the project to boost the uptake of referrals was particularly stressful for them.

Many LHWs expressed frustrations about their fixed salaries with no monetary or non-monetary compensation for additional programmes, such as eye care. In addition, their standard salary was often delayed with no communication or explanation of the reasons. As a result, they had to find other ways to feed their families and, in these circumstances, they could not do the extra visits to the patients to encourage them to take up referrals. There were also frustrations about hard working conditions and inability to receive medical supplies or replace damaged kits used in eye care.

### Patients’ health-seeking behaviour and barriers to referral uptake

#### Experience and knowledge of eye health

One of the main drivers of patient health-seeking behaviour was their perception of eye health problems. About half of the participants believed that minor issues were self-limiting or could be treated at home. Many preferred to wait and would only go to the hospital when the situation got worse. Women in particular prioritised the welfare of their family over their own eye health, which they thought was the best way to preserve respectability in the community:

*“I couldn’t go (to the hospital) because of the household chores...I did not have time.. who would respect a woman who does not prioritise the welfare of her children...” [Patient, Female, Non-adherent to Referral, Mansehra]*

Poor eyesight was perceived as part of the ageing process and many older people simply tried to adjust their lifestyle to accommodate visual impairments. For example, they tried to work in the daylight, read the Quran with large font and avoid activities involving near vision. Participants with poor vision tried to accommodate their impairment, usually until they had lost the ability to recognise their family members or faces. Often it was at this point that they would seek help. Even LHWs and LHSs themselves believed many eye-related problems were minor and could be addressed at any time:

*“…I know I have weak eyesight and I will get it checked [but] not today … after a few days. What is the worst that could happen? I would get to wear glasses. That is fine. This is the thinking that we people have, that we won’t be greatly affected by something so minor.” [Lady Health Supervisor]*

It was also reported that patients had little knowledge of the adverse consequences of delaying treatment, which contributed to the feeling that eye care was not a serious matter. Several participants emphasised a need for repeated messages about the risks of not attending eye examinations and referrals:

*“… they [LHWs] should make people understand, which they don’t do … Few people … care for themselves and there are many who don’t. That is not an issue for them. If [LHWs] provide awareness that this may lead to blindness and you may become disabled, then one would care.” [Patient, Male, Non-adherent to Referral, Mansehra]*

### Financial constraints

One of the key factors mentioned during the interviews with both patients and LHWs was the lack of financial resources to cover the costs of specialist eye care services and transport. This was, unsurprisingly, the reason for not taking up the referrals for most female patients. Another important factor was the lack of information about costs and services at the referral facilities. Many patients did not know what to expect at the secondary level hospitals. Some had had bad experiences in the past, including being turned away because they could not pay for medicines or surgical procedures. Patients explained that many of them were small-scale farmers, small-scale shopkeepers, daily wage earners and casual labourers and they could not afford fees charged by the hospitals coupled with the loss of their wages:

*“You know that if you came here [to the hospital], expenses are almost Rs1,000–1,200 and the loss of daily wages is 1,000 as well. So, the cumulative loss is more, which is unbearable for a person working on daily wages - how to make bread at home?” [Patient, Male, Non-adherent to Referral, Mansehra]*

As a result, the only way to address an eye problem for many patients was to use home – potentially harmful – remedies. For example, the use of surma, kagal and arqu-e gulab [which is essentially rose water] was frequently mentioned during the interviews as remedies to keep the eyes healthy and to treat eye allergies.

Most LHWs and LHSs themselves were unaware of how eye care hospitals worked: many had never visited an eye care department and did not know how much time patients needed for their referral or how much money the hospitals would charge:

*“… are there any charges in the DHQ [district hospital]?... I have no clue about their charges.” [Lady Health Supervisor]*

Given that the intention was to increase referrals and their uptake, not informing LHWs about such crucial service delivery details at the hospitals appears to have been a significant oversight of the training.

### Lack of trust in public sector hospitals

Several patients said that they did not trust the quality of services provided in public hospitals. Many had a view that eye surgeries carried out in a government hospital were of poor quality and that patients could go blind after the surgery in these public facilities, as one patient explained:

*“We have heard that they [public hospitals] did not operate successfully, people turned blind. Government hospitals take patients eyes out and patients become blind. Therefore, we avoid going to the government hospital.” [Patient, Female, Non-adherent, Mansehra]*

Another patient expressed similar views:

*“People these days visit private hospitals more frequently. Anyone who is doing better moneywise prefers private hospitals because they take better care of patients in private hospitals” [Patient, Female, Adherent to referral, Swabi]*

Some patients had fears of public hospitals irrespective of whether they personally had visited them or not. It seemed that some patients were more driven by their expectations of poor services rather than the actual experiences of these services:

*“… I got an accident at Karachi and … I had to bear 3–4 lakh. [US $1,875–2,500] in medical costs … If I go to Civil or Jinnah hospitals [public sector hospitals] they would have amputated my arm, because they do not have too much time. I got an operation [in a private hospital] for five hours; they removed body tissues from my leg and applied on the arm.” [Patient, Male, Non-adherent to referral, Mansehra]*

A number of patients said that they preferred visiting private hospitals because they could get all services in one place. Government hospitals were often lacking diagnostic equipment or laboratory services and had to send patients to private facilities, as one female patient explained:

*“… at the [public sector] hospital, they send you to Major Sb [a retired military doctor running a private clinic], so it is better to go to Major directly.” [Patient, Female, Non-adherent to referral, Mansehra]*

Patients further reported that public sector hospitals did not provide medicines, eyeglasses and they were not sure about the availability of doctors. At private not-for-profit hospitals, patients felt that doctors and medicines were always available; surgeries were successful and free and even lenses were provided free for certain categories of vulnerable patients. LHWs also noted that some government hospital staff had unhelpful attitudes, many hospitals were busy and lacking essential personnel, and patients – particularly old and vulnerable patients – did not feel comfortable in such facilities:

*“It is the attitude of staff and doctors. We feel that facilities … [should] test vision, prescribe medicines there and if he or she needs an operation, the doctor should properly guide them and refer them … and then at the … hospitals, these referred patients [should] be treated humanely and with good manners.” [Lady Health Worker, 15 Swabi]*

### Distance to hospitals and difficulties in travel

Another challenge reported by a number of patients was the distance to the health care facilities. Many patients found it very difficult to cover long distances to reach the main cities with health care facilities. This was particularly frequently reported in Mansehra district, where public hospitals were located in the city centre. They were difficult to reach for patients coming from far away areas with challenging terrain.

Travel was a particular challenge for older female patients, who had to find time, financial resources for themselves and a companion and get permission from their husbands or sons, who often did not take women’s health issues seriously, as one woman explained:

*“Sir, they [husbands] say as it is not a serious disease we are not permitted [to travel]. If they are not travelling with us, obviously alone women cannot go.” [Lady Health Worker 29, Mansehra]*

## Discussion

This study set out to assess the role of LHWs in improving access to eye health services in remote locations of Pakistan and specifically the uptake of referrals by patients with eye diseases. The study also explored the main drivers of patients’ eye health-seeking behaviour and barriers to eye care services. The study provides insights into the experiences of LHWs as the first points of contact for eye health. The findings draw on previous research on the nature and role of community health systems in facilitating the access to health care services for disadvantaged populations [19–22].

There are a number of key findings that have policy and programme implications. Overall, results show that LHWs and their supervisors were enthusiastic about the opportunities to be trained in primary eye care and be involved in providing eye care services to the underserved populations in their communities. However, similar to other research [23], our study demonstrates that training of primary eye care workers alone is not sufficient to increase patients’ access to specialist services. There are other elements of the health system that need to be adjusted to make the integration of the new function within the system a success. For example, in this context, the procurement of the LHW programme has not been changed to integrate the supply of basic ophthalmic equipment, such as torches or simple medicines and eye drops that are highly demanded by community members. The trained LHWs received their eye care kits only once, immediately after the training, and there was no provision for re-stocking or replacing the supplies. Similar problems were highlighted by a study in Tanzania, where primary health care workers were trained in eye care but the primary care facilities, where they worked, made no provisions for basic medicines in their procurement systems [23]. As a result, many stopped delivering eye care services, when their torches got broken or when they ran out of eye drops.

The training itself was generally well received, although there are questions as to whether the model of training of trainers (who themselves are not familiar with eye care) over three days followed by a one-day training for LHWs is sufficient to acquire the necessary knowledge and skills. The presence of an eye care specialist during the LHW training by their supervisors could potentially improve their understanding of eye care and particularly how secondary eye care facilities work. Another aspect of the training programme that will require attention is the lack of refresher courses for LHWs to update their knowledge and share their experiences with others. On the positive side, training of LHWs by their supervisors has possibly ensured a good level of supervision of their eye care work. In contrast to other studies [23, 24], the issue of lack of supervision has not been raised in this project. This seemingly effective approach can be replicated in other programmes, which involve primary or community level workers in eye care.

Another important factor raised during the interviews was the lack of clarity about LHWs’ responsibilities and what was expected from them. It seems that the LHWs were confused whether they were expected to treat patients with minor illnesses in the community or they were expected to refer everyone with an eye issue irrespective of how minor it was. It seems that the LHWs interviewed had different opinions about it and their practice varied. In this study we did not review any formal policies on the role of LHWs in eye care or what their responsibilities and competencies were. If the LHW programme continues to integrate the eye care function and is to be rolled out across the country, it would be critical to clearly articulate what LHWs could and could not do in eye care. LHWs need to be properly informed about how hospitals actually function in terms of structures and costs. It is also important that they are sufficiently trained to distinguish between the conditions that do and do not require a referral because a large number of referred patients with minor or self-limiting conditions can overwhelm already overcrowded and overstretched eye care hospitals [25, 26]. It can also undermine patients’ trust in the LHW programme, as patients will be unnecessarily travelling to faraway and expensive facilities.

It is also interesting that this programme was introduced in districts with relatively high coverage for cataract services, which means that patients do get to hospitals for at least serious sight threatening conditions such as cataracts. The problem highlighted in the recent RAABs was gender inequities, particularly in Swabi with a significantly lower cataract coverage among women. In this context one would expect that the primary purpose of the LHW’s role would be to identify female patients with cataracts and help them to receive the service. But this potential focus on women did not come out strongly in the interviews and it remains unclear whether LHWs have been specifically encouraged to find women with cataracts or not.

A number of factors affected the uptake of referrals by LHWs in this project. First, although the role of LHWs in maternal and child health and polio eradication campaigns has been well appreciated by their communities, some community members had doubts whether LHWs can be as effective in addressing more complex issues, such as eye diseases. It is possible, therefore, that some patients did not take seriously the LHWs’ advice to go to distant secondary facilities, where they will be expected to pay and wait for long hours. Second, there seemed to be a misunderstanding of what a LHW’s referral means for hospital services. It appears that both the LHWs and the patients expected a ‘special’ treatment at the hospital for those referred by the LHWs. It remains unclear whether this information was wrongly communicated to the LHWs during the training or it was their own interpretation of what their referral slip meant. It appears that many secondary-level providers were unaware of the LHW programme and their referrals. LHWs spent considerable time completing the slips, which ultimately did not have a required level of authority [27, 28]. As advocated by several participants in this study, improved engagement and communication between LHWs and hospital-based healthcare providers will be necessary to ensure that the referral pathways work effectively, and the referred patients receive the care they need.

In addition, many barriers to the uptake of referrals identified in this study were system-related and could not be addressed by the LHW referral programme alone. Similar to other rural contexts in South Asia [29, 30], we identified user fees and long distances to the facilities as major factors affecting patients’ decision-making. Also, in this specific context, the perceived poor quality and dissatisfaction with services in government hospitals were a major barrier. This finding is consistent with previous research that showed that overcrowded facilities with busy and unfriendly staff created significant barriers to the uptake of hospital referrals [25, 31].

Two types of barriers, however, could be potentially addressed by this programme: the lack of information about eye diseases and eye care services and the constraints faced by women who have to negotiate their travel and finances with their husbands or sons. Gender inequities in particular is an area where LHWs can be potentially effective [32] and their role in addressing these should be strengthened in future programmes. Gender inequities are deeply entrenched in Pakistan. The underlying gender structures which offer men numerous material benefits and decision-making authority contribute to women’s relatively lower uptake of health services [30, 33]. Previous studies in the country have shown that even those who have some source of income to facilitate travel to the hospital often choose not to – reflecting the values and ideals of being a good wife and putting the family first [33–35]. The government is aware of the problem and recognises that gender inequity is a key determinant of negative health outcomes among women [34, 36]. Indeed, the government recognises that more innovative service delivery models are required to improve women’s access to healthcare services [25, 36] but progress towards addressing the problem has been slow [37]. Using LHWs as case finders to identify women with cataracts and organising services closer to the communities, where women do not have to travel far, could be potentially an effective strategy to increase coverage with cataract services among women in these districts.

Finally, our findings suggest that the eye care programme in KPK increased pressures on LHWs who already had heavy workloads and grievances, such as late pay, to deal with. While training and deploying LHWs to identify and refer patients appeared to be generally acceptable, it also represented a significant challenge to the community health system. Some LHWs considered the additional responsibility to be a burden, which did not come with additional pay or support. In resource-limited contexts with overstretched and often inefficient health systems, adding more responsibilities to the workload of a fixed number of overworked and underpaid community workers should be very carefully managed, as this could potentially do more harm than good [12, 38].

This study has a number of limitations. First, the study was conducted in only two purposively selected districts of KPK province, with a small sample of patients, LHWs and managers, and do not necessarily reflect the experiences of other patients and health care providers outside the two districts. The range of patients, LHW and manager experiences may not be exhaustive when considering referral pathways and experiences in other parts of the province or the country. Second, while we selected participants to ensure that the different community health system configurations were included, we do not know to what extent the included LHWs and their supervisors are representative of others in the same district. Interviews with other community health workers and observations of everyday practice of facility-based health care providers may have revealed more nuanced health system dynamics and thereby identified other factors influencing the organisation, delivery and uptake of eye care services. A third limitation is that although we used a team of three researchers to independently code the transcripts, resources were not available to organize member check workshops with participants to review and comment on our analysis and interpretation of the data. Such engagement with participants would have provided a further check on the accuracy of our coding process and interpretation.

## Conclusions

Results from this study have shown that the training of community health workers in eye care was well received. However, even in programmes providing established access points for care such as LHWs, training in additional specialist services is not sufficient and does not result in improved access of patients if other parts of the health system are not strengthened. The role of LHWs in eye care in a given programmatic context should be articulated, and their responsibilities and competencies should be clearly defined. Pathways for referrals should be agreed and explicitly communicated to both the health care providers and the patients. Attention should be paid to how LHWs are remunerated and supported to prevent their overburden and demotivation. The role of LHWs could be made particularly effective and should be strengthened in providing patients with accurate information about eye diseases and services available. Further research specifically focused on women and surgical uptake is needed.

## Data Availability

The datasets generated and/or analysed during the current study are not publicly available due to the anonymity of the participants but are available from the corresponding author on reasonable request.

## Abbreviations

LHW: Lady Health Worker
LHS: Lady Health Supervisor
KPK: Khyber Pakhtunkhwa
PEC: Primary Eye Care
